# Perfecting Posterior Dental Simulacrum: Unveiling the Stamp Technique for Tooth Restoration

**DOI:** 10.7759/cureus.62640

**Published:** 2024-06-18

**Authors:** Manya Sonkar, Joyeeta Mahapatra, Aditya Patel, Manoj Chandak, Neha Pankey, Janhvi Bhosle

**Affiliations:** 1 Department of Conservative Dentistry and Endodontics, Sharad Pawar Dental College and Hospital, Wardha, IND; 2 Department of Pediatric and Preventive Dentistry, Sharad Pawar Dental College and Hospital, Wardha, IND

**Keywords:** aesthetic, accuracy, occlusal anatomy, posterior region, stamp technique

## Abstract

Dentistry is currently undergoing a phase where the pursuit of excellence has become continuous, and there is a rising demand for aesthetic standards, especially in the posterior region of the oral cavity. The “stamp technique” introduces a novel, straightforward method to restore carious teeth with unaltered occlusal structures using composite restoration. It effectively replicates the natural occlusal anatomy with remarkable precision. This method applies to preoperative carious teeth with preserved anatomy, minimally affected by carious lesions. Essentially, it involves creating an occlusal matrix from an undamaged occlusal surface of the tooth, aiming to achieve an accurate restoration resembling the natural tooth. This facilitates the restoration of the tooth’s natural contour and contact, ensuring precise functional occlusion. This procedure offers increased efficiency, requires less time while maintaining high accuracy, and reduces the time needed for finishing and polishing.

## Introduction

As amalgam restorations are less aesthetically pleasing and pose mercury-associated health dangers, posterior composite restorations are already taking their place. The development of minimally invasive restoration techniques, which emphasize preserving healthy tooth structure and using adhesive materials, further promotes composite resin restorations [1].

Composite restorations have become increasingly common among dentists; yet, to achieve a direct posterior composite restoration that is aesthetically attractive, one must possess remarkable dexterity and experience. Determining the cusp-fossa connection of teeth in direct composite restoration is complex, and occlusal harmony necessitates a substantial amount of clinical time in addition to operator competence. Final adjustments and polishing take longer than amalgam restoration [2,3]. To combine function and esthetic, Dr. Waseem Riaz presented an alternative approach called the "stamp technique" [4].

This novel stamping technique creates a small impression of the occlusal morphology by making an index first and then preparing the cavity. This technique proves useful in instances where there is no obvious cavity or destruction of tooth structure, but caries are visible on radiographs. The index is then placed into the last composite increment before curing to produce a positive duplicate. This approach mimics the pre-existing condition while offering the benefit of reducing the time needed to remove excess material and polish the restorations to the top of the form [5]. The other alternative technique for the stamp technique is the putty index technique.

## Case presentation

Case 1

**Figure 1:**
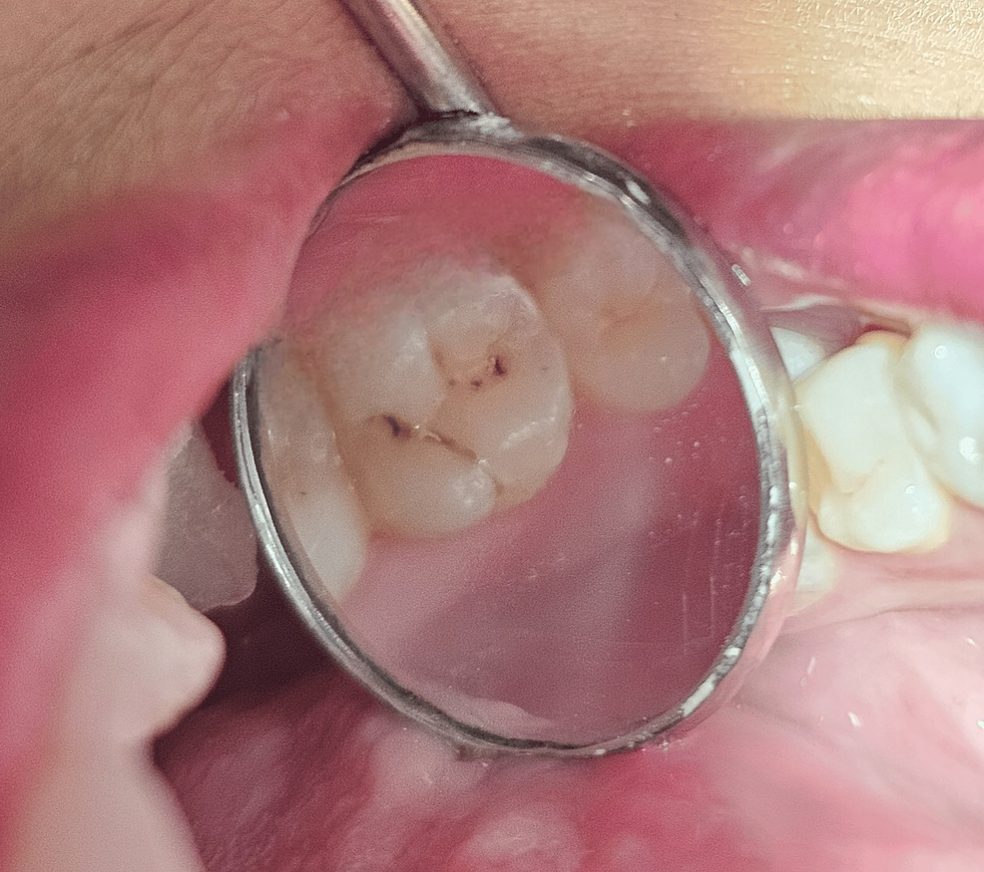
Preoperative clinical view of the tooth number 26

A 23-year-old female patient presented to the outpatient department (OPD) with a blackish discoloration in the upper left rear region of her jaw. Intraoral examination revealed pit and fissure caries in tooth number 26 (Figure 1). No evidence of marginal ridge involvement was found. As a result, it was decided to utilize composite resin and the stamp technique for reconstructing the decayed area.

**Figure 2:**
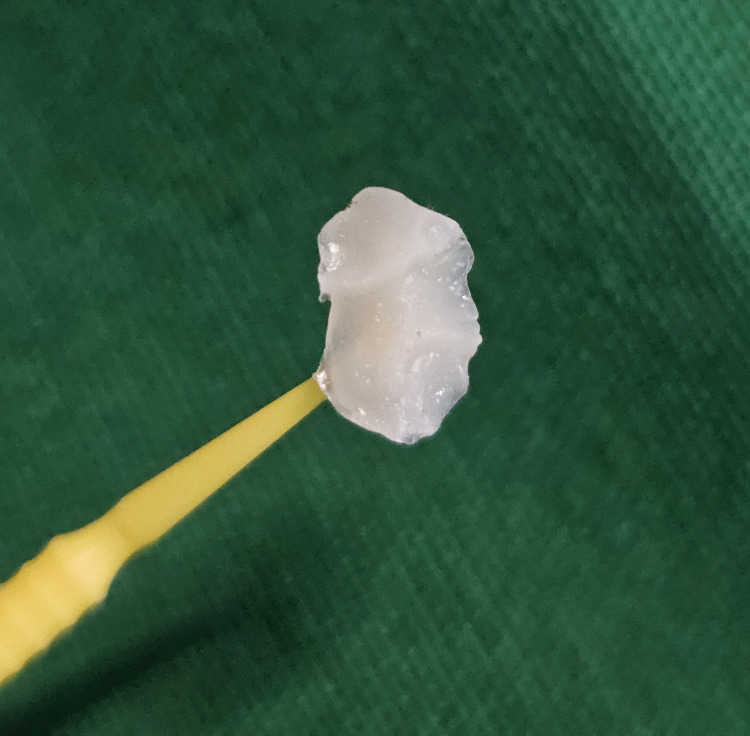
Composite stamp obtained from the occlusal surface of tooth number 26

**Figure 3:**
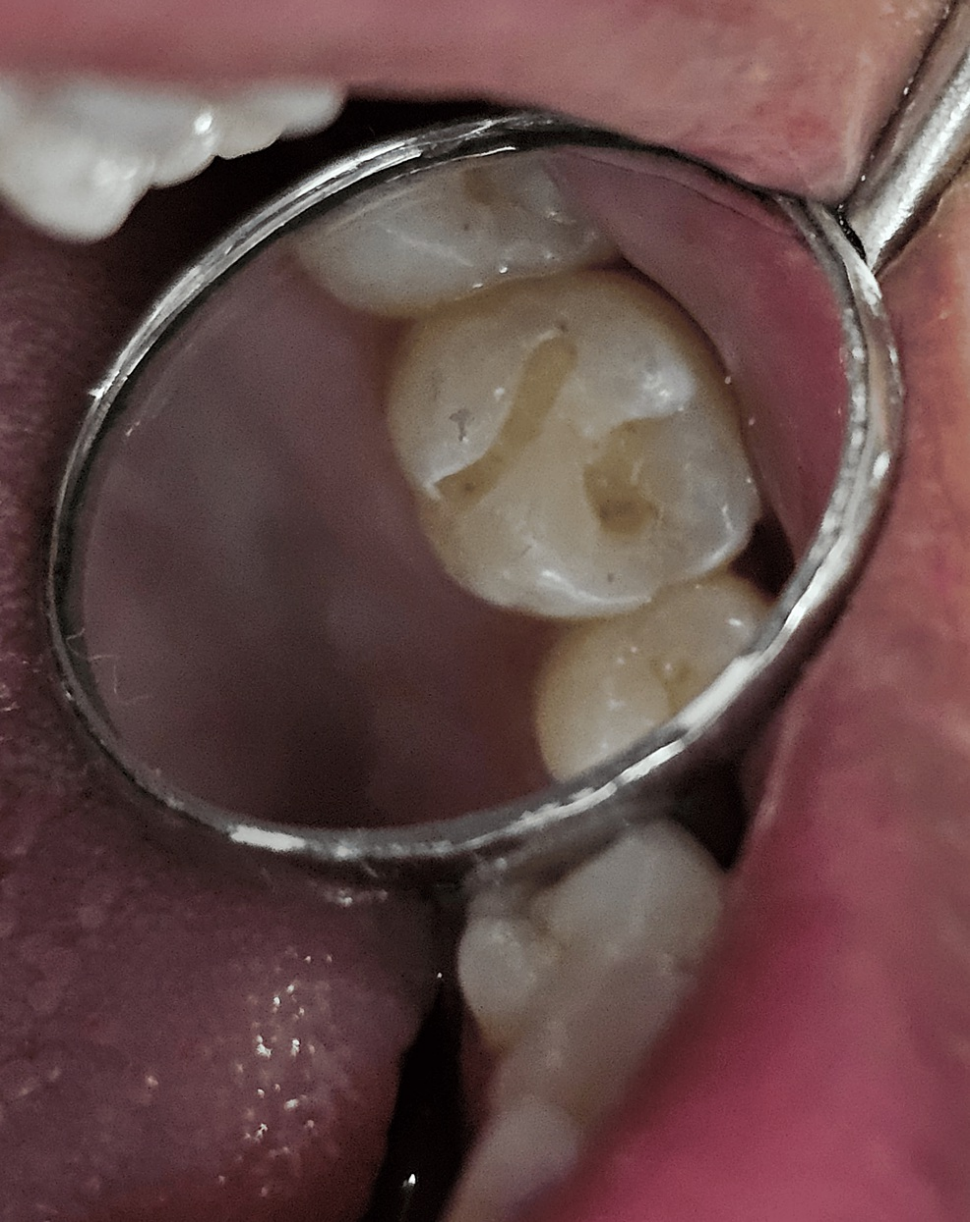
Class I cavity preparation done on the tooth number 26

After the tooth was isolated and dried, the occlusal surface was coated with a separating medium. Next, a flowable composite (3M Filtek Supreme) was applied to the tooth's unaltered occlusal surface to create the stamp. The tip microbrush was immersed up to 2 mm in the flowable composite to serve as a handle, followed by polymerization with the light-curing unit for 20 seconds (Figure 2). After excavation of the caries, a class I cavity was prepared (Figure 3).

**Figure 4:**
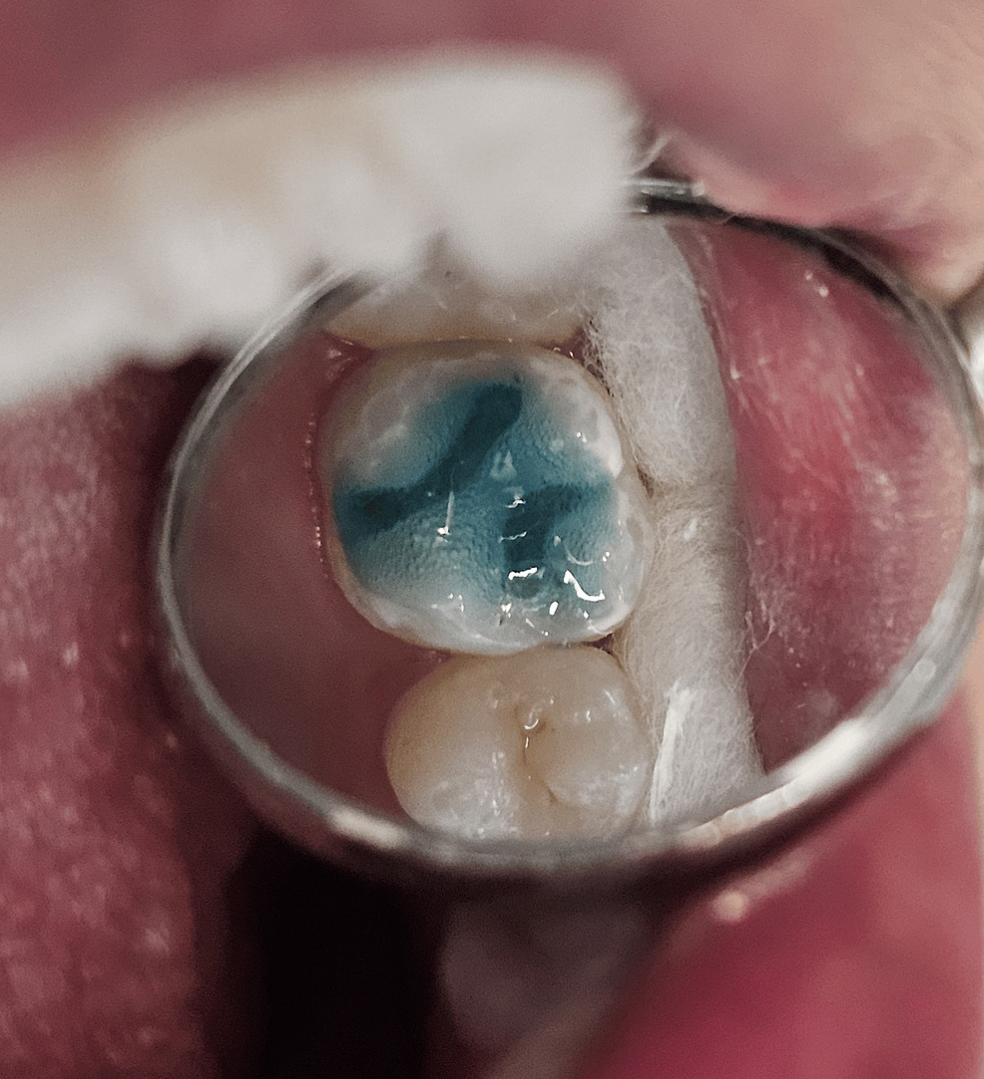
Etching done using 37% phosphoric acid on the prepared cavity of tooth number 26

The acid etching process was carried out for 10-15 seconds using 37% phosphoric acid (Ivoclar, Vivadent Eco-Etch etchant, Switzerland), and then the surface was rinsed and allowed to air dry (Figure 4). A 20-second light curing was carried out after applying the bonding agent (3M ESPE Single Bond Universal, USA).

**Figure 5:**
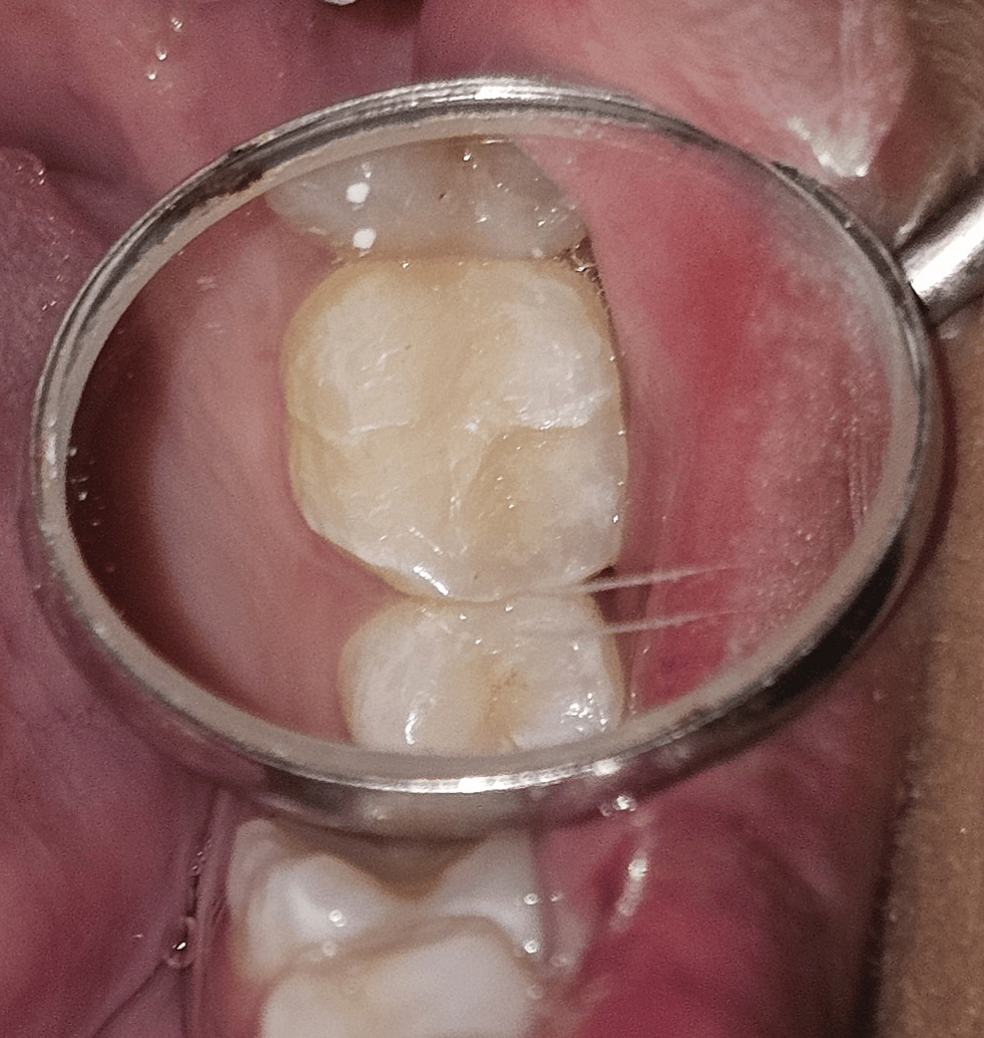
Composite restoration done on tooth number 26

Following that, the cavity was restored using the incremental composite resin procedure, with each layer being light-cured for 20 seconds and placed up to 1 mm below the occlusal surface. The occlusal surface was then covered with a piece of Teflon before curing the final layer of composite. Subsequently, the microbrush occlusal stamp was placed on top of the tape and lightly compressed. The composite was light-cured for 20 seconds after the excess material was removed. Very little polishing and finishing were done (Figure 5).

Case 2

**Figure 6:**
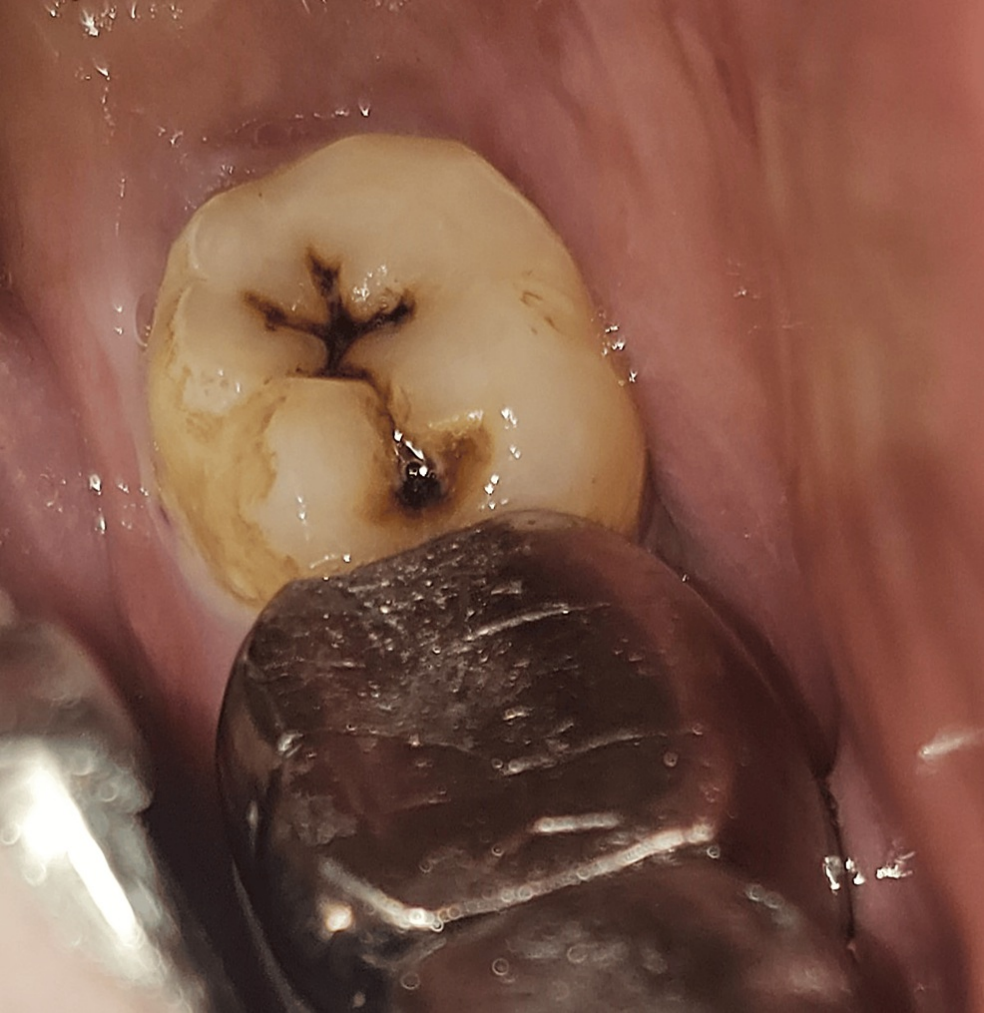
Preoperative clinical view showing pit and fissure caries on the occlusal surface in tooth number 38

**Figure 7:**
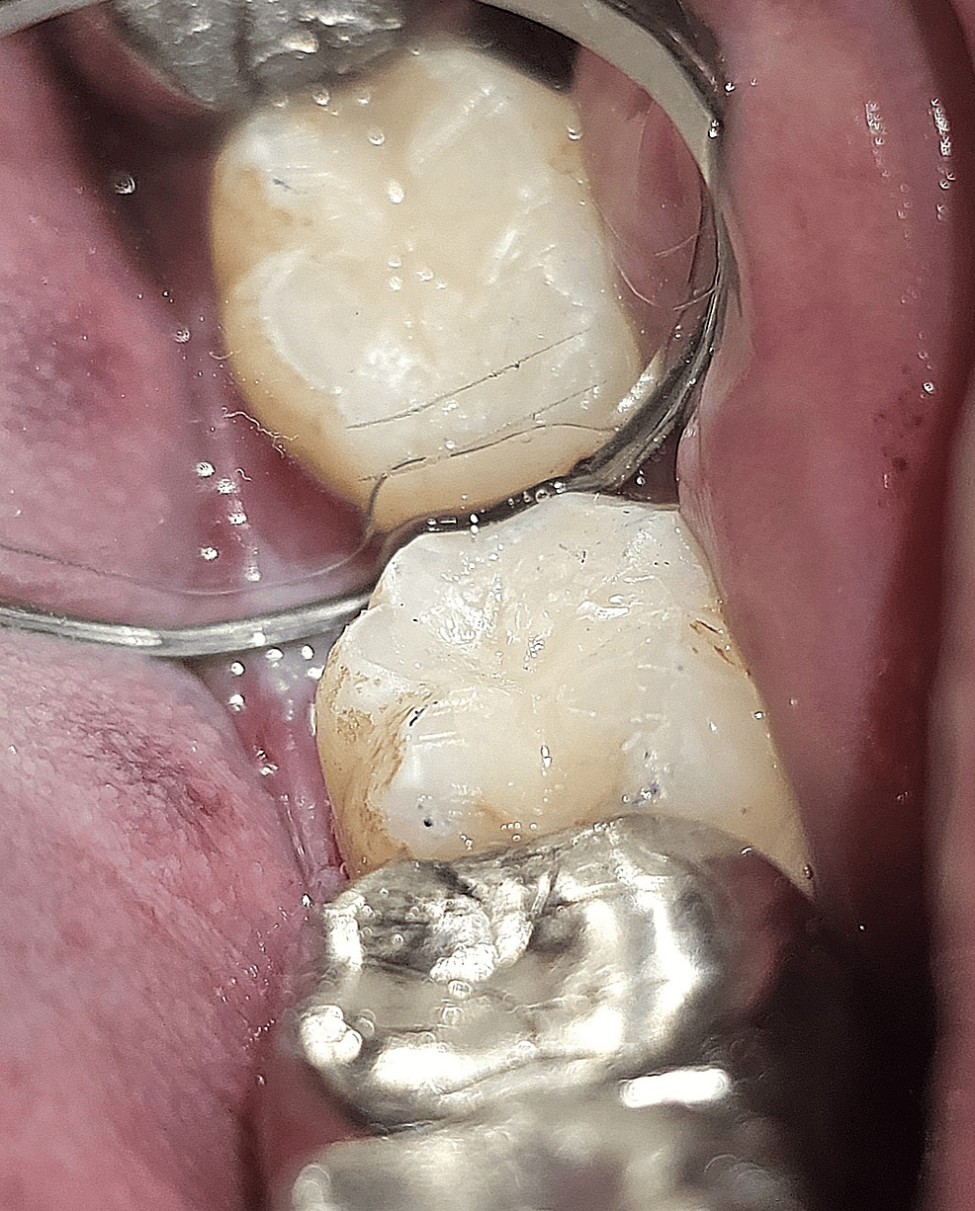
Composite restoration done on tooth number 38

A 58-year-old male patient reported to the OPD and complained of sensitivity in the lower left back region of the jaw (Figure 6). An intraoral examination revealed pit and fissure caries in tooth number 38. An intraoral peri-apical radiograph was employed to assess the caries extent, which revealed no signs of marginal ridge involvement. Consequently, composite resin and the stamp technique were used to reconstruct the decayed area. After the tooth was isolated, the same procedural steps of Case 1 were followed (Figure 7).

Case 3

**Figure 8:**
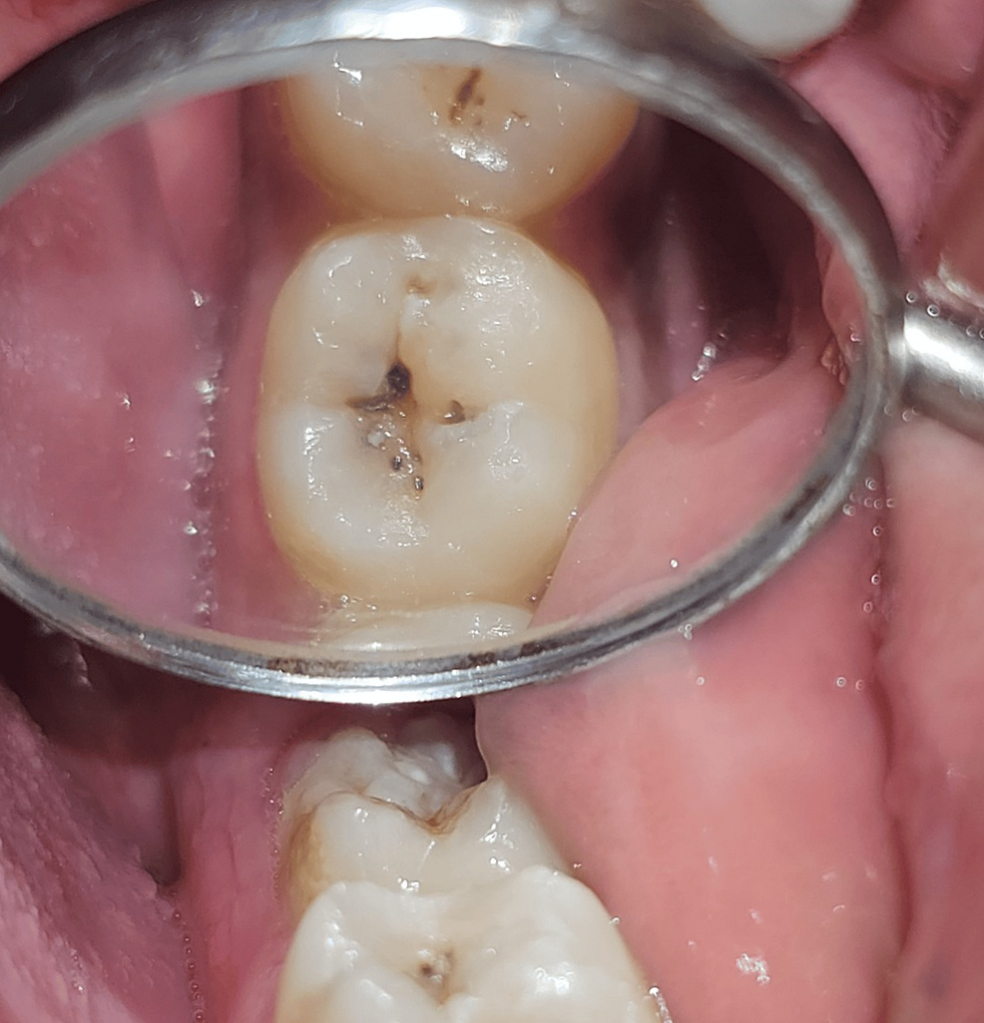
Preoperative clinical view showing pit and fissure caries on tooth number 37

**Figure 9:**
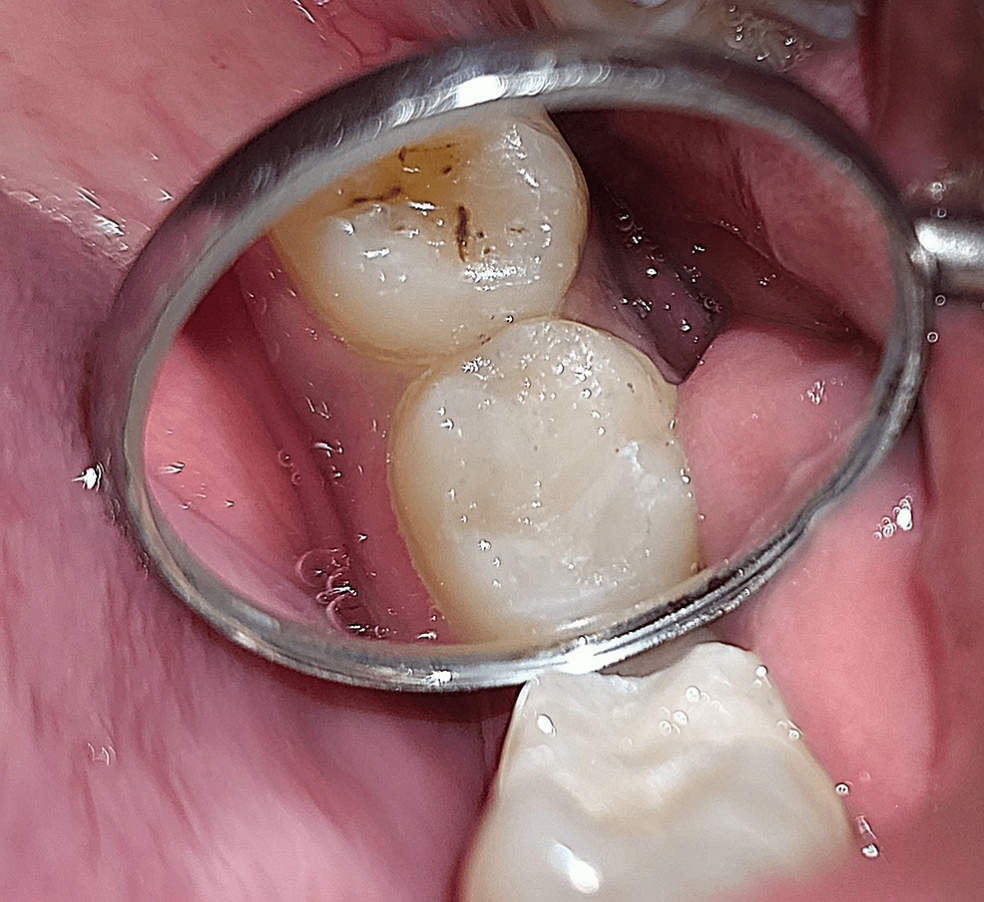
Composite restoration done on tooth number 37

A 22-year-old male patient reported to the OPD and complained of blackish discoloration in the lower left back region of the jaw (Figure 8). An intraoral examination revealed pit and fissure caries in tooth number 37. An intraoral peri-apical radiograph was utilized to evaluate the extent of caries, which showed no indication of marginal ridge involvement. Therefore, composite resin and the stamp technique were employed to reconstruct the decayed area. Subsequently, all procedures were performed as per Case 1 following tooth isolation (Figure 9).

## Discussion

Restoring the occlusal anatomy in anterior teeth mainly focuses on aesthetics. In contrast, in posterior teeth, it is crucial for preserving appropriate masticatory function and ensuring a correct cusp-fossa relationship with the opposing teeth. An unfavorable occlusion can lead to issues in the temporomandibular joint, centric occlusion, and the teeth, along with their supporting structures [6]. This case series outlines an easy method for achieving accurate anatomy and high-quality surface finish of direct posterior composite restorations using the “stamp technique” with flowable composite in minimal time [1]. Alternative materials that can be used for this purpose include polyvinylsiloxane impression material, pit and fissure sealants, bite registration paste, flowable gingival barrier, or pattern resin [7]. Flowable composite has been used for performing the mentioned cases because of its ability to resist deformation during its removal after impression making of the pit and fissures of the occlusal surface. The incremental layering technique should be employed to mitigate the primary drawback of polymerization shrinkage in composite restorations [8,9]. The stamp is utilized on the final layer to replicate the natural occlusal anatomy. Cling film can also be used as a barrier material; however, Teflon tape was the preferred option. Nevertheless, cling films are not required to be removed as they allow for the curing of the resin composite through it [10]. Furthermore, while using this technique, there is an evident reduction in the porosity in the finished restoration. This is because the stamp matrix applies pressure to the composite, which minimizes the formation of micro-bubbles and reduces oxygen interference with the polymerization of the final composite layer [11]. According to Vertuan et al., at four years of follow-up, small wear could be seen in the restoration, which was undertaken using the stamp technique. However, this wear was still within clinically acceptable levels [3]. According to Ramseyer et al., seven patients were assessed, and after a mean observation time of 40 months (40.8 ± 7.2 months), the overall outcome of the restorations was good [12].

The disadvantage of this technique is that it requires clinical and skill expertise to perform correctly. Additionally, it requires a flowable composite and a microbrush, which can be costly. Transparent acrylic resin or expired flowable composite can be used as alternatives to address this issue [13]. When all of the benefits and drawbacks of the stamp approach are taken into account, along with the incremental placement of composite restorations to reduce polymerization shrinkage, the stamp technique produces virtually flawless occlusal anatomy and improves workflow.

## Conclusions

An intact occlusal surface can be restored using the biomimetic occlusal stamp approach. In contrast to the manual method, it produces better outcomes in a shorter duration with fewer post-restoration filling adjustments. This treatment's main limitation is that it can only be applied to caries at the margins with preserved occlusal surfaces or pit and fissure cavities where the occlusal structure is still intact. For moderately widespread, irregularly shaped cavities that could result in increased shrinkage of the composite during polymerization, the conventional procedure can be applied; however, it takes additional time and requires operator skill.

## References

[1] Alshehadat SA, Halim MS, Carmen K, Fung CS (2016). The stamp technique for direct class II composite restorations: a case series. J Conserv Dent.

[2] Dilley DC, Vann WF Jr, Oldenburg TR, Crisp RM (1990). Time required for placement of composite versus amalgam restorations. ASDC J Dent Child.

[3] Vertuan M, Mosquim V, Guimarães GM, Obeid AT, Bombonatti JF, Ishikiriama SK, Furuse AY (2023). The stamp technique for direct restoration in a ICDAS 4 carious lesion: a 4-year follow-up. J Esthet Restor Dent.

[4] Mary G, Jayadevan A (2016). Microbrush stamp technique to achieve occlusal topography for composite resin restorations - a technical report. J Sci Dent.

[5] Guilherme J, Pompeu JG, Morais RC (2016). Occlusal stamp technique for direct resin composite restoration: a clinical case report. Int J Recent Sci Res.

[6] Gupta R, Thakur S, Goud KM, Fares KT, Jayasheel A (2021). A biomimetic approach for class 1 restorations using stamp technique: a case report. Int J Appl Dent Sci.

[7] Leon A, Ungureanu L, Caraiane A (2018). Transfer of occlusal morphology from dental laboratory to dental office through the stamp technique. Int J Appl Dent Sci.

[8] Mahapatra J, Nikhade P, Patel A, Taori P, Relan K (2022). Comparative evaluation of the efficacy of light-cured calcium hydroxide and a fourth-generation calcium silicate cement (TheraCal LC) as indirect pulp capping materials in patients with deep carious lesions: a randomized parallel-group clinical trial. Cureus.

[9] Mahapatra J, Nikhade PP, Patel A, Mankar N, Taori P (2024). Comparative evaluation of the efficacy of Theracal lC, mineral trioxide aggregate, and biodentine as direct pulp capping materials in patients with pulpal exposure in posterior teeth: a triple-blinded randomized parallel group clinical trial. Cureus.

[10] Tambake NJ, Tambake S, Gandhi N, Jadhav Y, Madhu K, Burad P (2017). Stamp technique - new perspective of aesthetic dentistry: a case report. IOSR J Dent Med Sci.

[11] Murashkin A (2017). Direct posterior composite restorations using stamp technique-conventional and modified: a case series. Int J Dent Res.

[12] Ramseyer ST, Helbling C, Lussi A (2015). Posterior vertical bite reconstructions of erosively worn dentitions and the “stamp technique”-a case series with a mean observation time of 40 months. J Adhes Dent.

[13] Nishad SV, Sharma U (2018). Stamp technique for posterior composite restorations-a case report. J Dent Med Sci.

